# Effects of administration route on uptake kinetics of ^18^F-sodium fluoride positron emission tomography in mice

**DOI:** 10.1038/s41598-021-85073-0

**Published:** 2021-03-09

**Authors:** Zaniah N. Gonzalez-Galofre, Carlos J. Alcaide-Corral, Adriana A. S. Tavares

**Affiliations:** 1 British Heart Foundation/University of Edinburgh Centre for Cardiovascular Science, Queen’s Medical Research Institute (QMRI), Little France Campus, 47 Little France Crescent, Edinburgh, EH16 4TJ UK; 2 Edinburgh Imaging, University of Edinburgh, Little France Campus, Edinburgh, EH16 4TJ UK

**Keywords:** Molecular imaging, Positron-emission tomography

## Abstract

^18^F-sodium fluoride (^18^F-NaF) is a positron emission tomography (PET) radiotracer widely used in skeletal imaging and has also been proposed as a biomarker of active calcification in atherosclerosis. Like most PET radiotracers, ^18^F-NaF is typically administered intravenously. However in small animal research intravenous administrations can be challenging, because partial paravenous injection is common due to the small calibre of the superficial tail veins and repeat administrations via tail veins can lead to tissue injury therefore limiting the total number of longitudinal scanning points. In this paper, the feasibility of using intra-peritoneal route of injection of ^8^F-NaF to study calcification in mice was studied by looking at the kinetic and uptake profiles of normal soft tissues and bones versus intra-vascular injections. Dynamic PET was performed for 60 min on nineteen isoflurane-anesthetized male Swiss mice after femoral artery (n = 7), femoral vein (n = 6) or intraperitoneal (n = 6) injection of ^8^F-NaF. PET data were reconstructed and the standardised uptake value (SUV) and standardised uptake value ratio (SUVr) were estimated from the last three frames between 45- and 60-min and ^8^F-NaF uptake constant (*K*_i_) was derived by Patlak graphical analysis. In soft tissue, the ^18^F-NaF perfusion phase changes depending on the type on injection route, whereas the uptake phase is similar regardless of the administration route. In bone tissue SUV, SUVr and *K*_i_ measures were not significantly different between the three administration routes. Comparison between PET and CT measures showed that bones that had the highest CT density displayed the lowest PET activity and conversely, bones where CT units were low had high ^8^F-NaF uptake. Intraperitoneal injection is a valid and practical alternative to the intra-vascular injections in small-animal ^18^F-NaF PET imaging providing equivalent pharmacokinetic data. CT outcome measures report on sites of stablished calcification whereas PET measures sites of higher complexity and active calcification.

## Introduction

The short lived radioactive tracer ^18^F-sodium fluoride (^18^F-NaF) is a positron-emitting radiotracer widely used in skeletal imaging since the 1960s due to its high affinity for bone hydroxyapatite matrix^1^. ^18^F-NaF has minimal protein binding allowing for fast soft-tissue clearance ﻿where nearly half of the injected radiotracer is taken up by the bones immediately after injection^2,3^. The remaining radiotracer is rapidly cleared from plasma and excreted by the kidneys^1^. ^18^F-NaF binds to areas of active bone formation, representing a marker of bone synthesis and blood flow^4,5^. Bone deposition of this radiotracer occurs via chemisorption, in which the OH^−^ ions in hydroxyapatite crystals are exchanged for ^18^F^−^ ions, converting hydroxyapatite to fluorapatite at sites of new bone formation^1,6^ reflecting active mineralising bone not only at active skeletal lesions and bone metastasis but also importantly on sites with vascular microcalcification^7,8^.

The use of ^18^F-NaF for bone metastasis imaging was fuelled by the development and installation of the first clinical Positron Emission Tomography/Computed Tomography (PET/CT) scanner, allowing high-resolution functional imaging with significantly greater sensitivity, specificity, and accuracy than conventional planar bone scintigraphy with bisphosphonates^9,10^. Recently, this old PET radiotracer has been re-purposed as a biomarker of active calcification in coronary atherosclerosis^11,12^, an unfavourable event in the history of atherosclerosis that predicts cardiovascular morbidity and mortality^13^. These vascular calcifications are a hallmark of atherosclerotic burden, and several biological mechanisms, including the release of calcifying extracellular vesicles, alterations in local microenvironment and increased mineralization^14,15^. Studies have shown that the ^18^F ions of ^18^F-NaF bind to the surface of nanocrystalline hydroxyapatite and the intensity of ^18^F-NaF uptake is higher for the smaller sized crystals^16^, therefore supporting its use as a marker of active cardiovascular microcalcification. With expanding number of animal models of vascular calcification^17–22^ and the advent of high-sensitivity/high-resolution preclinical PET systems^23,24^, studies with ^18^F-NaF in mice are likely to increase in coming years. Furthermore, since the mid-1980s, the effort to understand mammalian biology and the need to study disease models have caused the mouse to become the animal of choice. Given that most human genes have a related mouse gene, this rodent can be used as a platform for simulating many human diseases^25,26^. Therefore, it is unsurprising that many mouse models of human cancer are also available, many of which present with bone metastasis that could be imaged with ^18^F-NaF PET technique^27–30^.

Like most PET radiotracers, ^18^F-NaF is typically administrated via intravenous injection. Although routine in humans, intravenous injections in small animals, namely mice, can be more challenging, since they require proper animal-handling techniques and partial paravenous injection is common because of the small calibre of the superficial tail veins, thus leading to technical challenges on small-animal PET research^31^. Furthermore, reproducible intravenous injection is not always possible for longitudinal studies or for sequential or multiple-tracer injection studies due to residual activity from previous injections^32,33^.

In this study we aimed to investigate the feasibility of using intra-peritoneal route of injection of ^8^F-NaF to study calcification in mice by looking at the kinetic and uptake profiles of normal soft tissues and bones versus intra-vascular injections.

## Materials and methods

### Radiochemistry

^18^F-NaF was prepared using a cyclotron and a FASTlab synthesiser (GE Healthcare), using commercially available cassettes and was formulated in saline (0.9% w/v).

### Animals

All experimental procedures were performed in accordance with the relevant guidelines and regulations. Experiments reported here were carried out in compliance with the ARRIVE guidelines, approved by the local University of Edinburgh animal ethics committee, and authorized by the Home Office under the Animals (Scientific Procedures) Act 1986. Nineteen 10- to 17-week-old Swiss mice (SD-1 males, 35.8 ± 3.6 g) were used for this study. All animals were housed and maintained at the Edinburgh Preclinical Imaging facility, University of Edinburgh, UK under standardised 12 h light:12 h dark conditions with food and water available ad libitum. Animals were split into three experimental groups depending on the administration route of the radiotracer (7 for femoralartery injection studies, 6 for femoralvein injection studies and 6 for intra-peritoneal injection studies).

### Surgical vessel cannulations

For the femoral vein and femoral artery experimental groups, anaesthesia was induced and maintained using isoflurane (2–2.5%, oxygen 0.5 L/min, nitrous oxide 0.5 L/min) during the surgical procedure. Polyethylene catheters (PE10) filled with heparinized (20 IU/mL) saline were inserted into the left femoral artery or vein with the help of a stereomicroscope and securely fastened with ligatures (6-0 silk thread). Catheters were held in place with surgical glue and the animals were then transferred to the PET/CT scanner (nanoPET/CT, Mediso, Hungary) for all subsequent imaging procedures. Given that the cannulation was done into the left femoral artery or vein, the right femur and the right tibia were used to obtained bone measurements and are referred in the results as femur and tibia.

### PET/CT data acquisition and reconstruction

Mice received an intra-vascular or intra-peritoneal injection of ^18^F-NaF as follows: intra-arterial administration (8.68 ± 3.94 MBq, mean ± SD, n = 7) via femoral artery; intra-venous administration via femoral vein (7.43 ± 4.69 MBq, mean ± SD, n = 6); and intra-peritoneal administration (7.85 ± 2.18 MBq, mean ± SD, n = 6). Immediately post-radiotracer administration, a 60 min whole-body emission scan was obtained using a 1:5 coincidence mode (where each detector module is in coincidence with five opposing ones, and is equivalent to maximum field of view of 102 × 102 × 94.7 mm). Then, a CT scan was acquired (semi-circular full trajectory, maximum field of view, 360 projections, 35 kVp, 170 ms and 1:4 binning) for attenuation correction. PET data was reconstructed into 6 × 30 s, 3 × 60 s, 2 × 120 s, 10 × 300 s frames using Mediso’s iterative Tera-Tomo 3D reconstruction algorithm and the following settings: 4 iterations, 6 subsets, full detector model, low regularisation, spike filter on, voxel size 0.4 mm and 400–600 keV energy window. PET data were corrected for randoms, scatter and attenuation.

### Processing and analysis of PET single organ time-activity curves

Reconstructed scans were imported into PMOD 4.0 software (PMOD Technologies, Switzerland) and volumes of interest (VOIs) were manually drawn around organs and bones of interest using the CT data with exception of the kidneys and the vena cava, where the PET image was used. Radioactivity in the blood was estimated using the blood pool in the vena cava. Time-activity curves (TACs) were generated for each organ and standardised uptake values (SUV, g/mL) were calculated according to Eq. (1):

1 $$\mathrm{SUV}(\mathrm{g}/\mathrm{mL})=\frac{\mathrm{radioactivity\; concentration\; in\; volume\; of\; interest }\;(\mathrm{MBq}/\mathrm{mL})}{\frac{\mathrm{injected\; dose \;}(\mathrm{MBq})}{\mathrm{animal\; weight }\;(\mathrm{g})}}$$

Kinetic modeling was performed using Patlak analysis (t* = 16 min) to estimate the influx constant (*K*_*i*_). The reference tissue VOI used for Patlak modelling was the blood pool VOI in the vena cava. The use of the inferior vena cava is a well stablished approach when modelling PET mice data. SUV and SUVr averages were taken from the last 3 frames between 45 and 60 min.

### Data analysis

Results are presented as standard error of the mean (SEM). Statistical analyses were performed using Prism, version 8.4.3 (GraphPad Software Inc.). Differences in physiologic parameters among the experimental groups of different administration routes were compared by two-way ANOVA with Tukey’s multiple comparison post hoc test. A *P* value of less that 0.05 was considered statically significant.

### Ethical approval

All methods and animal experiments carried out in this study were performed in compliance with the ARRIVE guidelines and regulations, severity protocols, and with ethical permission from the University of Edinburgh Animal Welfare and Ethical Review Body and the UK Home Office under the Animals (Scientific Procedures) Act 1986.

## Results

### ^18^F-NaF kinetics in soft tissue is different depending on the administration routes during perfusion phase but are similar during uptake phase

**Figure 1:**
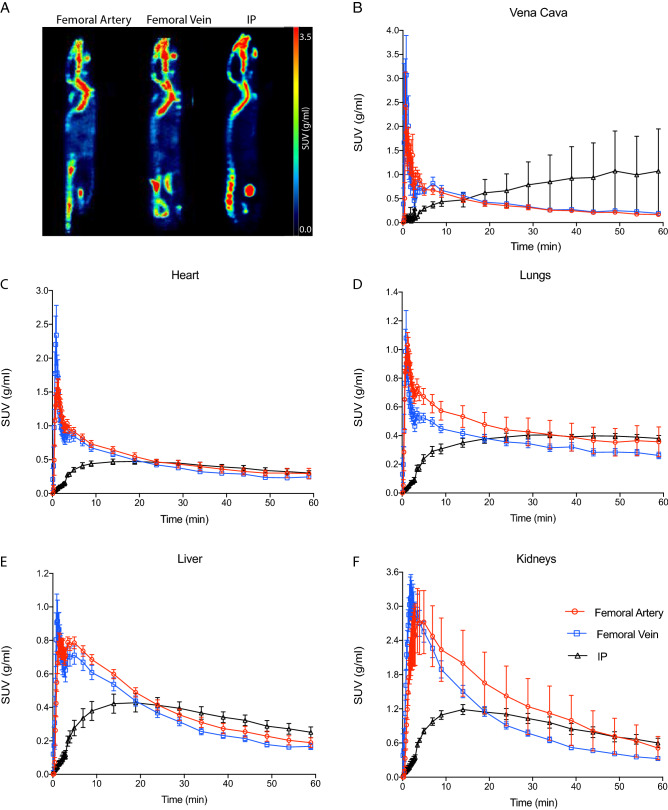
Representative examples of biodistribution of ^18^F-sodium fluoride (NaF) after femoral artery, femoral vein and intra peritoneal injections presented as average sagittal section images between 45 and 60 min post-injection (**A**). Mean time-activity curves of the vena cava (**B**), heart (**C**), lungs (**D**), liver (**E**) and kidneys (**F**) after femoral, artery, femoral vein or intraperitoneal (IP) injection. Error bars represent one SEM. *SUV* standardised uptake value.

Representative ^18^F-NaF images of intra-vascular or intraperitoneal injections are shown in Fig. 1A. Intense radioactivity can be observed at sites of calcification, i.e. the skeletal system of naïve mice. Time courses of ^18^F-NaF activity in the vena cava, heart, lungs, liver and kidneys are shown in Fig. 1B–F. Following intravascular injection, ^18^F-NaF rapidly cleared from the blood pool, heart, lungs and liver followed by the kidneys (Fig. 1B–F). After intraperitoneal injection, radioactivity was initially intense in the peritoneal cavity followed by a slow clearance onto the tissue (Supplementary Fig. S1). Although tracer uptake was slower for the intraperitoneal route, activity in most organs reached concentrations comparable to the intra-vascular routes of administration within 15–20 min after injection.

### PET outcome measures in bone tissue are not affected by the administration route

**Figure 2:**
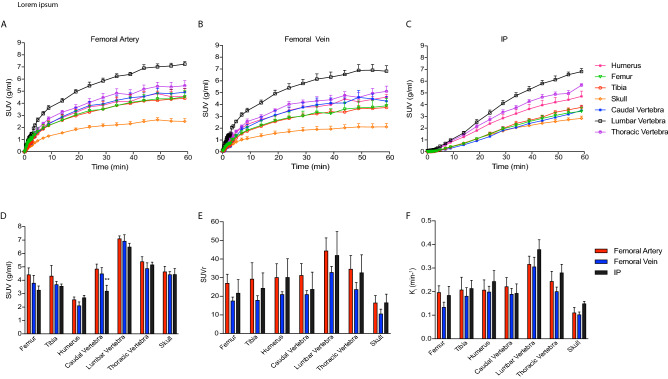
Bone mean time activity curves after femoral artery (**A**), femoral vein (**B**) and IP injection (**C**) of ^18^F-NaF. Error bars represent one SEM. Mean standardised uptake value; SUV (**D**), standardised uptake value ratio relative to blood pool; SUVr (**E**) and influx constant; *K*_*i*_ (**F**) of several bones after femoral artery, femoral vein and intraperitoneal (IP) ^18^F-NaF administration. Error bars represent one SEM. 2-way ANOVA with multiple comparisons and Tukey’s post-hoc test was used to calculate *P* values. (**P = 0.0087 femoral artery vs IP administration route).

Time courses of ^18^F-NaF activity in several bones are shown in Fig. 2A–C, where the injected compound accumulates in the bone over time. The lumbar vertebra showed the highest ^18^F-NaF uptake (≈ 7 g/ml) whereas the skull showed the lowest (2 g/ml–3 mg/ml). This was true regardless of the route of injection, where ^18^F-NaF uptake was similar between intra-vascular or intraperitoneal injection where highest to lowest ^18^F-NaF uptake was an indicator of higher to lower calcification in those regions. Furthermore, group averages (50–60 min) for SUV, SUVr and *K*_*i*_ for the different bones of interest showed no significant differences between the three administration routes (Fig. 2D–F), apart from the SUV in the caudal vertebra measured following femoral artery administration versus intraperitoneal injection (two-way ANOVA with multiple comparisons and Tukey’s post-hoc test **P = 0.0087).

### ^18^F-NaF PET measures bone mineralisation activity independent of the radiotracer administration route, while CT reports on established bone density

**Figure 3:**
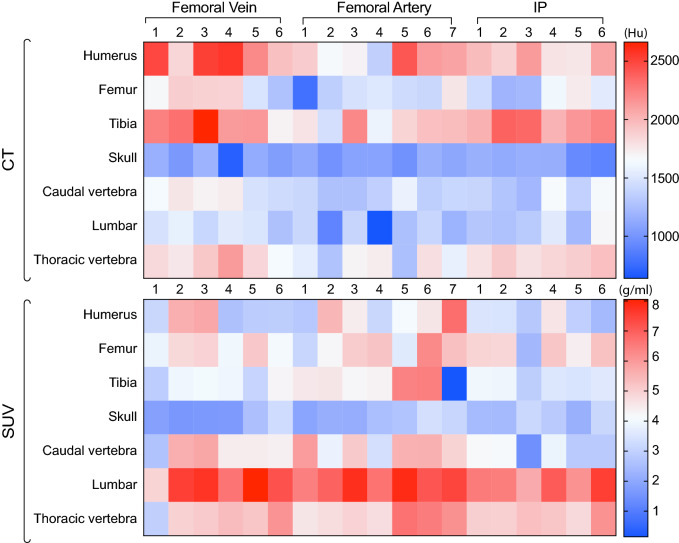
Heatmap comparing CT values versus PET uptake of ^18^F-NaF for each bone after femoral artery, femoral vein or intraperitoneal (IP) injection. Each column represents a mouse replicate per administration route. CT values are expressed as hounsfield units (HU) and standardised uptake values (SUVs) as g/ml.

Figure 3 shows a heatmap comparing CT measures (expressed in Hounsfield Units, HU) and PET measures (expressed as SUV) following intra-vascular and intraperitoneal injections. The bones with highest CT density are the humerus, femur and tibia. These same bones display the lowest PET activity. Conversely, data shows that in the caudal, lumbar and thoracic vertebrae the CT units were low while the SUVs were high. There was no correlation between CT HU and PET SUV measurements (Supplementary Figure S2).

## Discussion

The present study investigated the feasibility of intraperitoneal injection as an alternative to the conventional intra-vascular injection for small animal ^18^F-NaF PET. Our results indicate the similarity and equivalence of intra-vascular and intraperitoneal injection. Previous studies on rodents using intraperitoneal injection of ^18^F-fludeoxyglucose (^18^F-FDG) showed similar results between intravenous and intraperitoneal administration routes^34–37^.

Reproducible intravenous injection is not always possible for studies that require continuous or repeated measures over prolonged periods of time due to damage to the vein, or multiple-tracer injection studies because of residual activity. In contrast, intraperitoneal injection is a practical alternative because it is faster and easier to perform and has been proved to be highly reproducible and with reduced stress to the animal, although care should be taken to avoid accidental intestinal or other organs administration during intraperitoneal injections of substance^34,35^. Our results indicate that all investigated routes of administration provide similar pharmacokinetic parameters and comparable ^18^F-NaF uptake and distribution within 60 min after injection. Nevertheless, the distribution of intraperitoneally injected ^18^F-NaF in various organs is slower because ^18^F-NaF has to diffuse across the peritoneal membrane and the absorption is via the portal system^38^. Therefore, a simplified yet robust protocol for quantification of ^18^F-NaF PET data in mice studies would be to administer the radiotracer via intraperitoneal injection and scan the animals after 45–60 min post-injection.

Our data comparing CT measures and PET measures for the three administration routes showed CT-positive, PET-negative regions in long cortical bones (humerus, tibia and femur) and CT-negative, PET-positive regions in smaller but more trabecular and complex bones (caudal, lumbar and thoracic vertebrae). Owning to ^18^F-NaF uptake mechanisms^11–13,15,16^, these observations indicate that the PET signal maps out active bone mineralisation while CT reports on established bone density. Furthermore, data indicate that the binding of ^18^F-NaF is dependent on surface area where long bones have less ^18^F-NaF activity and more complex surface structures^16^. Although this duality of the PET/CT responses in highly mineralised tissue of the skeletal system is newly reported in mice in this paper, previous studies looking at the use of ^18^F-NaF in aortic microcalcification have shown that PET measures identify regions of active calcifications whereas CT measures indicate area of stablished calcification^39,40^. Our preclinical data reported in this paper show this contrast was also present in the mouse skeleton and that could be robustly measured with all injection routes tested. Therefore this result provides confidence that SUV measurements using dynamic PET and intraperitoneal injections are able to recapitulate important biological features with equal confidence as traditional intravascular routes.

## Conclusion

Our results show that intra-peritoneal injection is a valid and practical alternative to the intra-vascular injections in small-animal ^18^F-NaF PET imaging and provides equivalent pharmacokinetic data. Furthermore, our data show that CT outcomes report on sites of stablished calcification whereas PET measures sites of higher complexity and active calcification.

## Supplementary Information

Supplementary Information
