# Supplementary material for: Effects of administration route on uptake kinetics of 18F-sodium fluoride positron emission tomography in mice
Source: Sci Rep. 2021 Mar 9;11:5512. doi: 10.1038/s41598-021-85073-0 (PMC7970902; doi:10.1038/s41598-021-85073-0)
Supplement: Supplementary file 1 — Supplementary Information [file 41598_2021_85073_MOESM1_ESM.docx]

**Supplementary file**

**Effects of administration route on uptake kinetics of ^18^F-sodium fluoride Positron Emission Tomography in mice**

Zaniah N. Gonzalez-Galofre^1,2^, Carlos J. Alcaide-Corral^1,2^, Adriana A. S. Tavares^1,2*^

^1^ British Heart Foundation/University of Edinburgh Centre for Cardiovascular Science, Queen’s Medical Research Institute, Little France Campus, Edinburgh, EH16 4TJ, UK.

^2^ Edinburgh Imaging, University of Edinburgh, Little France Campus, Edinburgh, EH16 4TJ, UK.

**Name and address for correspondence:**

Adriana Tavares, PhD

Queen’s Medical Research Institute (QMRI)

47 Little France Crescent

EH16 4TJ, Edinburgh, UK

**Email:** [adriana.tavares@ed.ac.uk](mailto:adriana.tavares@ed.ac.uk)

**
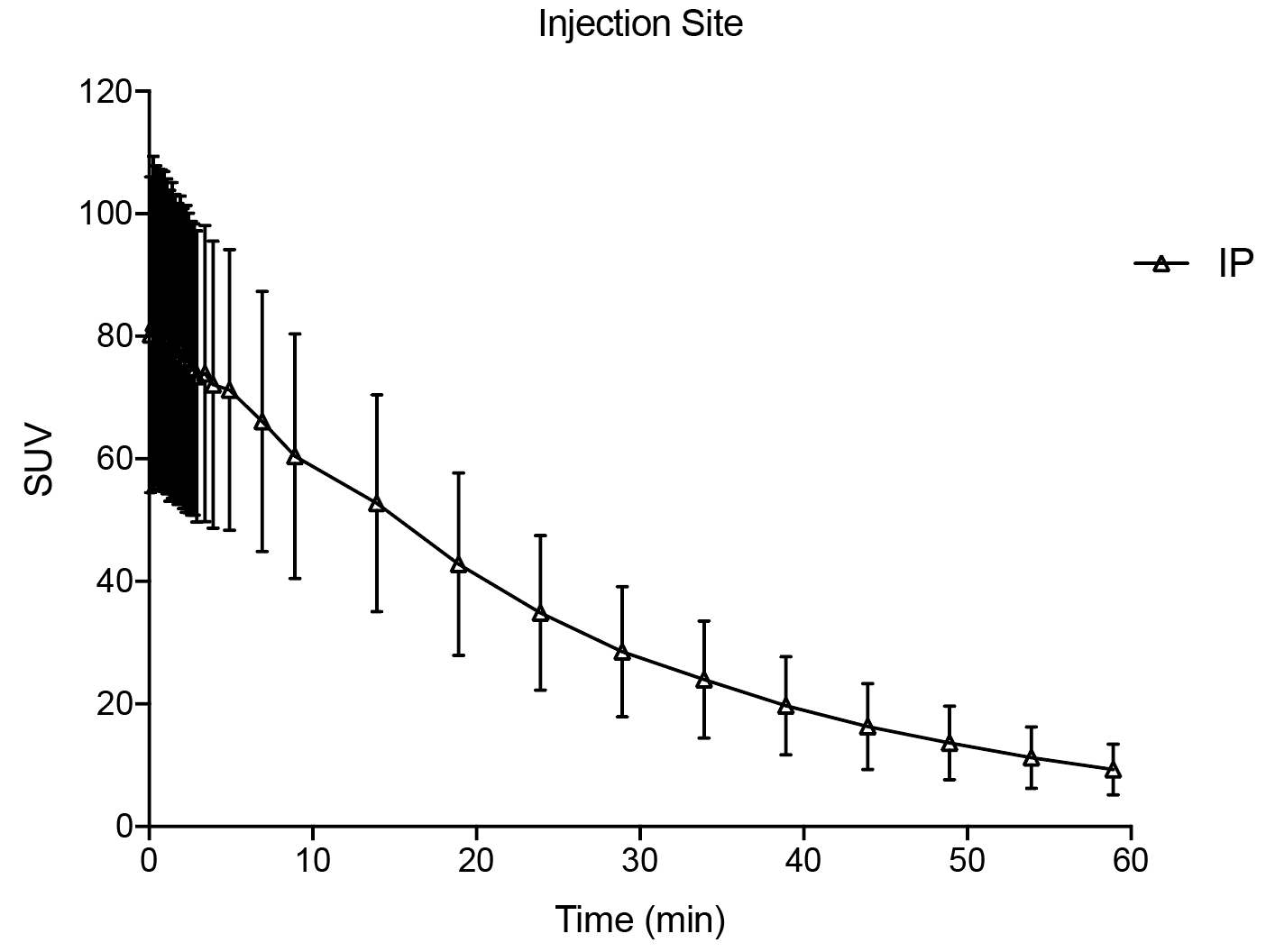
**

**SUPPLEMENTARY FIGURE 1.** Mean time-activity curve of the injection site after intraperitoneal (IP) injection. Error bars represent one SEM.

**
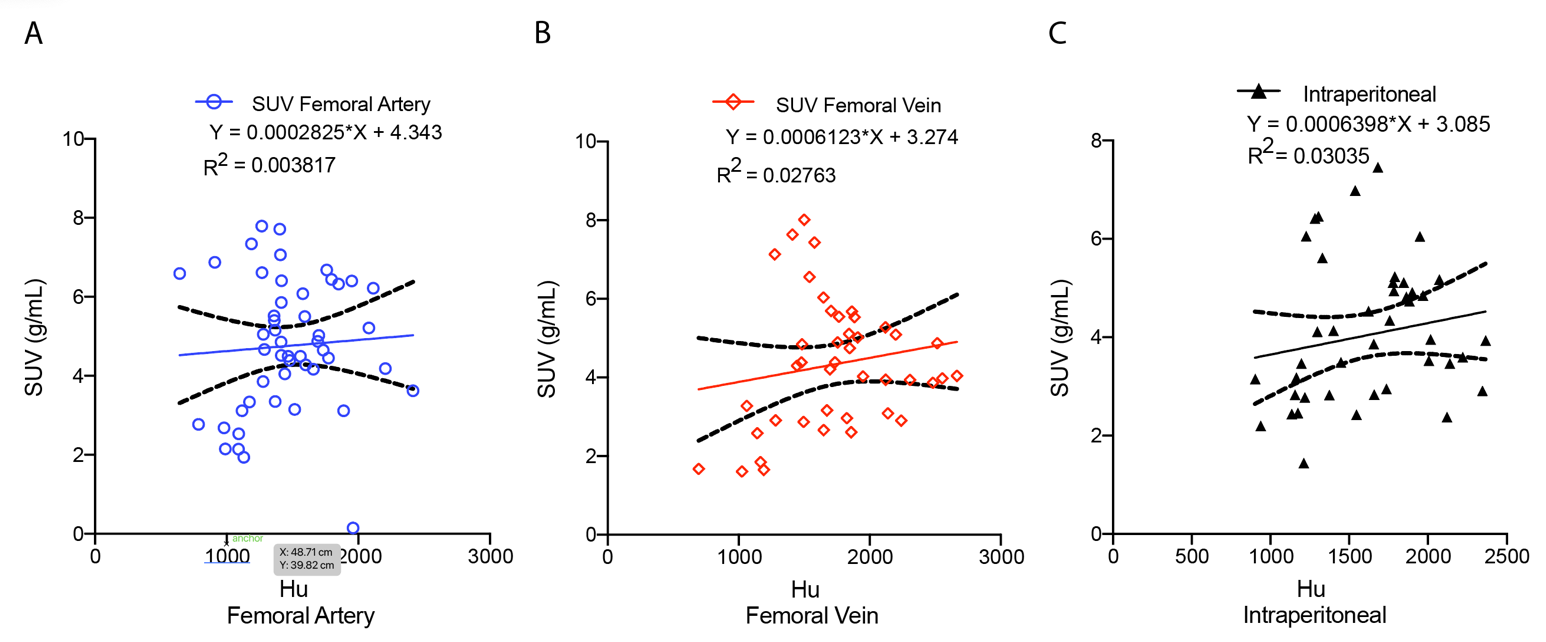
**

**SUPPLEMENTARY FIGURE 2.** Correlation analysis between CT HU and PET SUV measurements in femoral artery **(A)**, femoral vein **(B)** and intraperitoneal injections **(C)**. No correlation was found between the measurements in neither of the three injection routes.
